# Synthesis, Structure and Iodine Adsorption Properties of a Ni Cluster-Based Supramolecular Framework

**DOI:** 10.3390/molecules30050989

**Published:** 2025-02-21

**Authors:** Jingyi Qiu, Linxia Tang, Ziang Nan, Luyao Liu, Qing Li, Wei Wang, Zhu Zhuo, Dongwei Zhang, Yougui Huang, Liangliang Zhang

**Affiliations:** 1 College of Chemistry and Materials Science, Fujian Normal University, Fuzhou 350002, China; xmqiujingyi@fjirsm.ac.cn (J.Q.); xmtanglinxia@fjirsm.ac.cn (L.T.); 2 Xiamen Key Laboratory of Rare Earth Photoelectric Functional Materials, Xiamen Institute of Rare Earth Materials, Haixi Institutes, Chinese Academy of Sciences, Xiamen 361021, China; nanziang@fjirsm.ac.cn (Z.N.); xmliuluyao@fjirsm.ac.cn (L.L.); xmliqing@fjirsm.ac.cn (Q.L.); wangwei@fjirsm.ac.cn (W.W.); yghuang@fjirsm.ac.cn (Y.H.); 3 School of Microelectronics, Northwestern Polytechnical University, Xi’an 710072, China; 4 Strait Institute of Flexible Electronics (SIFE, Future Technologies), Fujian Normal University and Strait Laboratory of Flexible Electronics (SLoFE), Fuzhou 350117, China

**Keywords:** Ni cluster-based framework, porous, *π–π* stacking, active sites, iodine capture

## Abstract

The capture of radioactive iodine (129I or 131I) is of significant importance for the production of nuclear power and the treatment of nuclear waste. In recent years, crystallized porous materials have been extensively investigated to achieve highly effective adsorption of radioactive iodine. Herein, by using the hydrothermal method, a Ni cluster-based framework (**1**) was successfully constructed through a self-assembly process. Driven by the *π–π* stacking interactions between *π*-electron-rich benzimidazole groups, [Ni_5_S_6_] clusters stack in a lattice, forming a porous framework with proper channels, rendering compound **1** as an ideal adsorbent for iodine. Compound **1** delivered a capability of iodine adsorption (2.08 g g−^1^ and 560 mg g−^1^ for gaseous and solution iodine, respectively) with stable cyclability.

## 1. Introduction

With the gradual development of modern industrialization, the amount of traditional fossil fuels has reduced rapidly, leading to prominent problems with natural environment deterioration and global warming. Currently, efficient and clean energy sources, such as nuclear reactors and various nuclear power plants taking advantage of atomic nuclei, radioactivity and radionuclides, have been explored [1]. The waste of these nuclear energy sources, containing radionuclides ^127^Xe, ^85^Kr, ^235^U, ^137^Cs, ^90^Sr, ^99^Tc, ^79^Se, ^129^I and ^131^I, for instance, if not disposed of properly in a timely manner, will cause long-term adverse effects on human health and the ecological environment [2,3,4].

^129^I and ^131^I, as isotopes of common stable iodine (^127^I), directly affect the biological metabolic system once ingested by human bodies, and may lead to hypothyroidism or even thyroid cancer, genetic mutation and other health problems in serious cases [5,6]. Consequently, the development of innovative methods to achieve effective molecular I_2_ adsorption is of paramount importance.

Nowadays, crystalline porous materials (CPMs) are exploited to extract I_2_, including zeolites, metal–organic frameworks (MOFs), covalent organic frameworks (COFs), porous organic cages (POCs), metal–organic cages (MOCs) and supramolecular assemblies. Novel CPMs are urgently needed for molecular iodine separation due to their high adsorption capacity, excellent selectivity and high chemical stability within a certain temperature range [7,8,9,10,11,12]. Among them, metal cluster-based frameworks, as extensively studied CPMs, have demonstrated notable iodine adsorption performance. For example, a series of Al_8_ aluminum–oxygen molecular ring-type clusters with an iodine adsorption amount of 50.3 wt% of AlOC-15-AlOC-25 were reported [13]. Sujittra et al. have synthesized a dinuclear complex [Ni_2_(IDA)_2_(dpe)(H_2_O)_4_], with excellent iodine adsorption in hexane with a removal efficiency of 98.8% [14]. Thus, the frameworks of metal clusters possess significant advantages regarding iodine adsorption and show broad application prospects in the fields of environmental treatment and industrial wastewater treatment.

As a typical electron acceptor, molecular iodine can bind to CPMs containing electron-donating atoms or groups such as nitrogen, oxygen, sulfur, carbonyl, hydroxyl and aromatic rings through an electron transfer process, realizing more efficient adsorption behavior of I_2_ by CPMs [15]. Enhancement of I_2_ adsorption capacity can be achieved by introducing nitrogen atoms from aromatic rings, forming interactions with guest I_2_ through either hydrogen bonds (NH····I) or electron transfer between the nitrogen atoms and I_2_ [16,17,18]. Moreover, complexes with aromatic rings could enhance the adsorption behavior of iodine as well due to the presence of the large *π–π* conjugation system of the aromatic rings [19]. Therefore, the development of metal cluster-based frameworks incorporating the active groups mentioned above holds promise for efficient iodine molecular adsorption.

Herein, by using ligands with benzimidazole groups, a Ni^2+^-based cluster (compound **1**) was synthesized with the structure of a monolithic three-dimensional network through *π–π* stacking interactions. Since the crystal structure of compound **1** is porous and possesses mounts of electron-donating sites, including nitrogen atoms and benzimidazole groups, it demonstrated effective performance of dissolved I_2_ and I_2_ vapor capture. In addition, compound **1** showed excellent reusability after circulation several times with no significant loss of adsorption ability for I_2_.

## 2. Results and Discussion

### 2.1. Synthesis and Characterization of Compound ***1***

**Figure 1:**
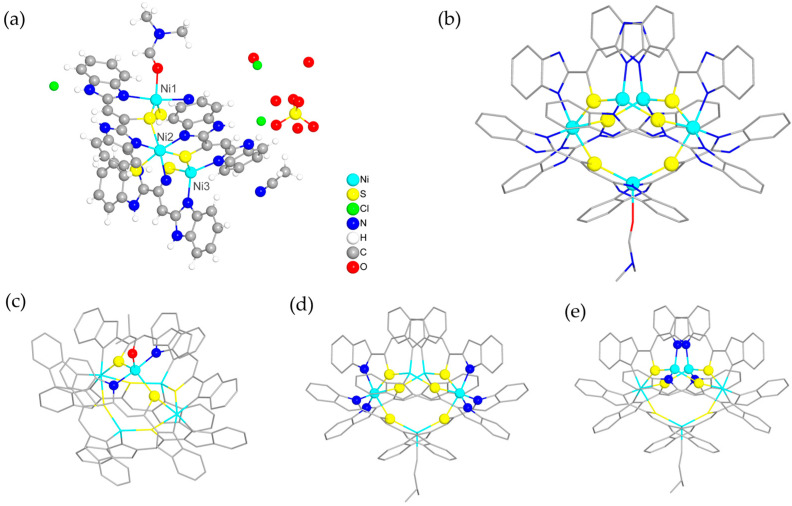
(**a**) The asymmetric unit of compound **1**. (**b**) Bonding pattern of the [Ni_5_S_6_] cluster. (**c**) Fivefold coordination pattern of the Ni1 atom. (**d**) Sixfold coordination pattern of the Ni2 atom. (**e**) Quadruple coordination pattern of the Ni3 atom. (All hydrogen atoms are omitted for clarity in (**b**–**e**).)

**Figure 2:**
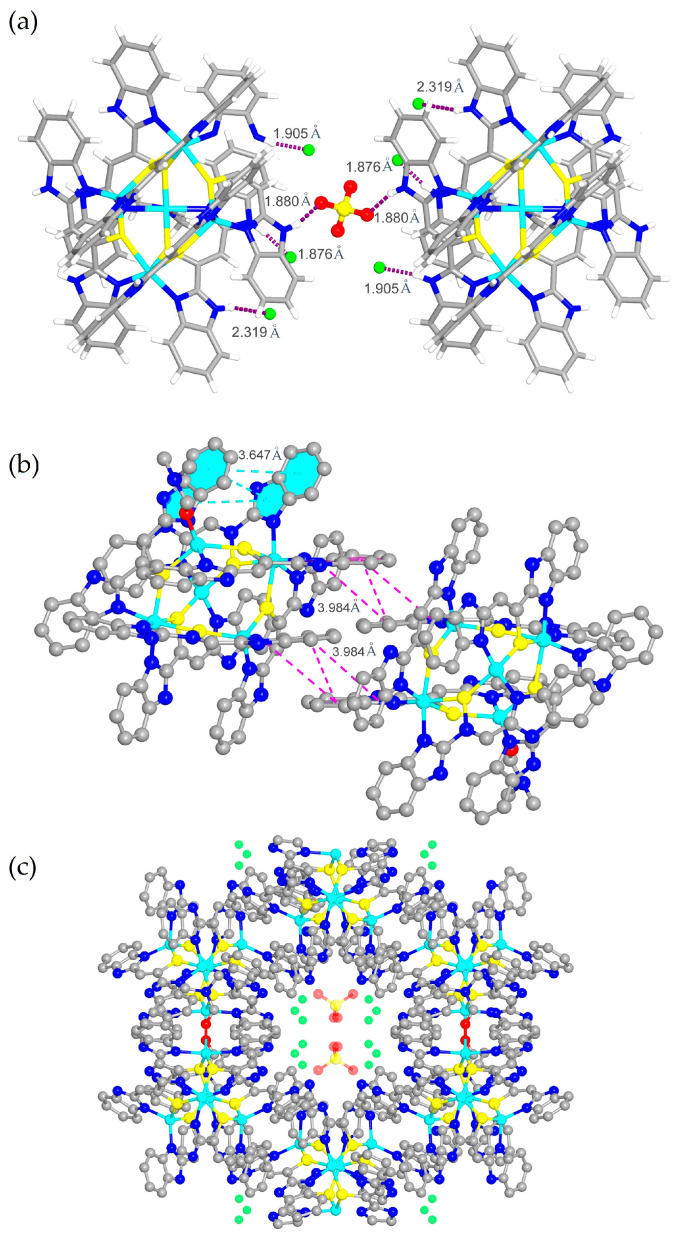
(**a**) Hydrogen bonds within compound **1**. (**b**) The π–π stacking interactions of compound **1** (the blue ones are intramolecular forces; the pink ones are intermolecular forces). (**c**) Porous in a three-site network. Color codes for atoms: green spheres, Ni; blue spheres, N; yellow spheres, S; red spheres, O; grey spheres, C.

The self-assembly of ligand 2-(2-benzimidazolyl)ethanethiol (L) (Figure S1a) with NiCl_2_·6H_2_O in a mixed solvent of acetonitrile, *N*,*N*-dimethylacetamide (DMF) and trimethylamine solution affords glossy black crystals of compound **1**. Single crystal X-ray analysis (Table S1) shows that compound **1** crystallized in the monoclinic *C*2/*_c_* space group. During the reaction, two ligands (L) are condensed into a dimer (L_1_) with coordination sites of sulfur and nitrogen atoms (Figure S1b). The asymmetric unit of compound **1** contains two-and-a-half Ni^2+^ ions, three deprotonated L_1_ (L_1_-H^+^), half of a DMF molecule, a quarter of SO_4_^2−^, one-and-a-half Cl^−^ ions, a half of acetonitrile and three-and-a-half H_2_O molecules (Figure 1a), resulting in the chemical formula [Ni_10_(L_1_-H^+^)_12_(DMF)_2_](SO_4_)Cl_6_(CH_3_CN)_2_(H_2_O)_14_ of compound **1**. The coordination environments of the Ni^2+^ ions are different (Figure 1b). Each Ni1 is five-coordinated with one oxygen atom from one DMF, one nitrogen atom and one sulfur atom from two deprotonated L_1_ (Figure 1c). Ni2 is six-coordinated with three deprotonated L_1_ through their sulfur and nitrogen atoms while Ni3 atom is four-coordinated with two deprotonated L_1_ (Figure 1d,e). It has to be noted that not all of the nitrogen atoms from deprotonated L_1_ within the cluster are engaged in coordination, which could be donated as active sites for guest molecule capture. As shown in Figure 1b, the cluster [Ni_5_(L_1_-H^+^)_6_(DMF)], donated as [Ni_5_S_6_], is a hollow structure containing five Ni and six S atoms with the average length of ~2.33 Å for Ni-S bonds. With the association of hydrogen bonding and *π–π* stacking interactions, [Ni_5_S_6_] clusters are linked into a three-dimensional network of compound **1**. [Ni_5_S_6_] clusters and the anions SO_4_^2−^ and Cl^−^ undergo alternating pileups through hydrogen bonds (Figure 2a) and the neighboring [Ni_5_S_6_] clusters are connected by *π–π* interactions. The distance of *π–π* interactions within the [Ni_5_S_6_] cluster is measured to be ~3.647 Å (blue), while the distance of *π–π* interactions between adjacent [Ni_5_S_6_] clusters is ~3.984 Å (pink) (Figure 2b). Thus, channels of ~6.389 Å in diameter are created through the process of stacking and are subsequently filled with solvent and ClO_4_^−^ ions (Figure 2c). As determined by the PLATON software package, the total void volume (*V*_void_) is 2205.6 Å^3^ per unit cell, which is 20.7% of the unit volume. However, the evaluation of the surface area of compound **1** failed because it is stabilized by non-covalent *π–π* interactions between clusters and CH_3_CN molecules located at the pores. As a result, it is unstable under vacuum despite its porosity.

**Figure 3:**
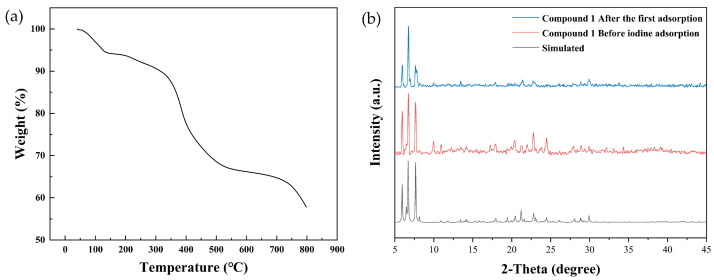
(**a**) TG analysis of compound **1**. (**b**) PXRD pattern of compound **1**.

Thermogravimetric (TG) analysis under N_2_ flow of compound **1** (Figure 3a) revealed that the first stage of weightlessness began from room temperature to ~100 °C, which might be caused by the weight loss of water and acetonitrile in the structure. After an extended period of gradual volatilization of DMF molecules from ~100 °C to ~330 °C, a rapid weight loss occurred as the structure began to collapse. This was attributed to the fracture and decomposition of compound **1** under high-temperature conditions. As the temperature continues to rise to ~500 °C, another stage of weight loss was observed. The experimental powder X-ray diffraction (PXRD) of compound **1** was characterized, which is consistent with the simulated pattern derived from the single-crystal structure, confirming the purity of the synthetic sample (Figure 3b).

### 2.2. Iodine Adsorption of Compound ***1***

**Figure 4:**
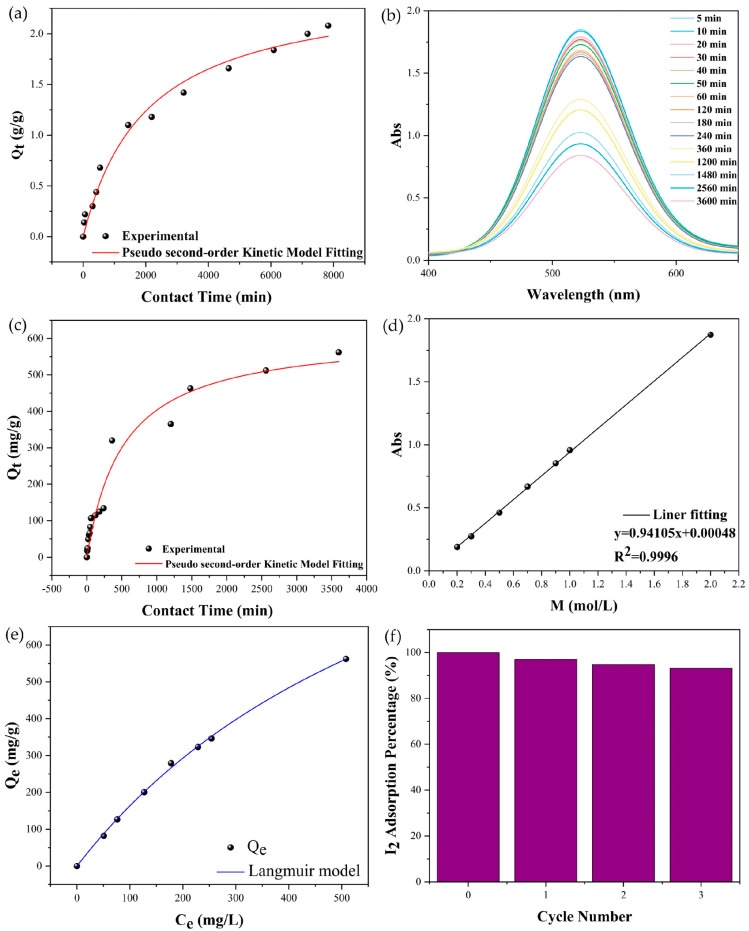
(**a**) Time-dependent iodine vapor absorption curve of compound **1**. (**b**) Time-dependent UV-Vis spectra of I_2_-cyclohexane solution with compound **1** as adsorbent. (**c**) Kinetic study of iodine adsorption by compound **1** in I_2_-cyclohexane solution at room temperature. (**d**) Standardized plot of absorbance of I_2_-cyclohexane solution (λ = 523 nm) versus I_2_ concentration. (**e**) Isothermal adsorption model fitting for iodine adsorption by compound **1** at room temperature. (**f**) Graph showing the recyclability of compound **1** for dissolved iodine adsorption. (Abs: the absorbance in UV spectroscopy tests; Q_t_: the amount of iodine adsorbed at a given time; Q_e_: the amount of iodine adsorbed at the equilibrium time; C_e_: the concentration of the iodine/cyclohexane solution).

We used stable molecular isotope ^127^I instead of radioactive iodine isotopes ^129^I and ^131^I in actual nuclear waste for iodine adsorption experiments, taking advantage of the identical chemical properties of the iodine isotopes [20]. Adsorption of gaseous iodine at 70 °C and atmospheric pressure were performed. Crystals of compound **1** were exposed in iodine vapor (Figure S2). Weight increases in exposed compound **1** (0.010 g) were recorded at different times to evaluate its performance, as shown in Figure 4a. With the increase in contact time, the iodine vapor adsorption uptake of compound **1** was promoted gradually. The uptake reached ~2.08 g g^−1^, remaining unsaturated after ~7800 min. Fitted by the quasi-secondary kinetic model (correlation coefficient *R*^2^ = 0.98289), the adsorption rate k and equilibrium adsorption capacity *Q*_e_ of compound 1 for vapor iodine were calculated to be 1.99757 × 10^−4^ g g^−1^ min^−1^ and 2.48412 g g^−1^, respectively (Table S2).

The adsorption performance for iodine dissolved in a solution of compound **1** was explored. To avoid interference caused by polar solvents [21], the non-polar cyclohexane was chosen to dissolve iodine. Specifically, crystals of compound **1** (0.010 g) were immersed in 20 mL iodine–cyclohexane solution (2 mol/L). The absorbance was measured using ultraviolet and visible (UV-Vis) spectrophotometry to analyze the adsorption degree of iodine (Figure 4b). At the initial stage, the adsorption rate was fast because of the strong driving force of high-concentration iodine diffusion to the adsorption site of compound **1**. With the decrease in adsorption sites along with the iodine concentration, the adsorption rate of compound **1** became slow after ~240 min. The uptake of compound **1** almost saturated at 4300 min with the amount of ~560 mg g^−1^. Meanwhile, the color of the crystals changed from the initial glossy black to dull black (Figure S3) and the purple color of the iodine–cyclohexane solution faded (Figure S4), which proved the successful adsorption of dissolved iodine. Similar to the vapor iodine, the adsorption process of dissolved iodine could be better described by the pseudo-second-order kinetic model compared to the pseudo-first-order kinetic model (Figure 4c). The correlation coefficient *R*^2^ was 0.96219, and the saturated adsorption capacity was 614.60865 mg g^−1^ through calculation (Table S3).

The adsorption capacities of compound **1** at the same temperature (25 °C) for different concentrations of iodine in cyclohexane (0.2, 0.3, 0.5, 0.7, 0.9, 1.0, 2.0 mmol/L) were explored. The absorbance at 525 nm of iodine solutions of varying concentrations was initially evaluated using ultraviolet and visible (UV-Vis) spectrophotometry and the resulting curve of absorbance variation with iodine concentration was subjected to a curve-fitting analysis. The equation of the fitted curve was y = 0.94105x + 0.00048 (*R*^2^ = 0.9996), and the iodine concentration in cyclohexane corresponding to different absorbances can be obtained from this linear relationship (Figure 4d). With the increase in initial concentration, *Q*_e_, which was further fitted using the Langmuir equation and Freundlich equation, it increased gradually (Figure 4e). As shown in Table S4, the *R*^2^ value of the Freundlich equation was smaller than that of the Langmuir equation, indicating the more reasonable adsorption model for the Langmuir and the occurrence of single-molecular-layer iodine adsorption on the surface of compound **1**.

The gaseous and dissolved iodine uptake capacity of compound **1** is comparable to that of some promising adsorbents (Table S5) [22,23,24,25,26,27,28,29]. I_2_@**1** was immersed into methanol to explore the desorption property of iodine. The color of the methanol solution gradually changed from colorless to dark yellow (Figure S5), suggesting the release of iodine. After three adsorption–desorption cycles, the relative adsorption capacity for gaseous iodine was still above 90% (Figure 4f), which makes compound **1** a promising absorbent for iodine adsorption, with the advantages of low cost and environmental friendliness.

## 3. Discussion

**Figure 5:**
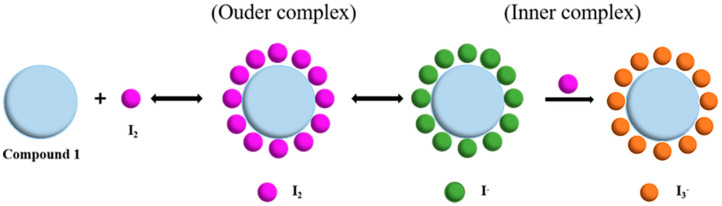
Steps in the formation of polyiodide ions during iodine adsorption.

The uncoordinated nitrogen and sulfur atoms possess lone-pair electrons. Thus, compound **1** can be considered as a Lewis acid. These atoms can form charge transfer complexes with iodine molecules via an *n* → σ* interaction [30,31]. When iodine molecules were adsorbed onto the crystals of compound **1**, a charge transfer process involving the lone pair orbitals (*n*) of the uncoordinated N atom occurred, forming an external charge complex with the antibonded σ* orbitals of iodine molecules (Figure 5) [32,33,34,35]. This external charge complex subsequently underwent leptogenesis to form an internal charge complex, which may further adsorb additional iodine molecules, resulting in a polyiodide ion complex.

**Figure 6:**
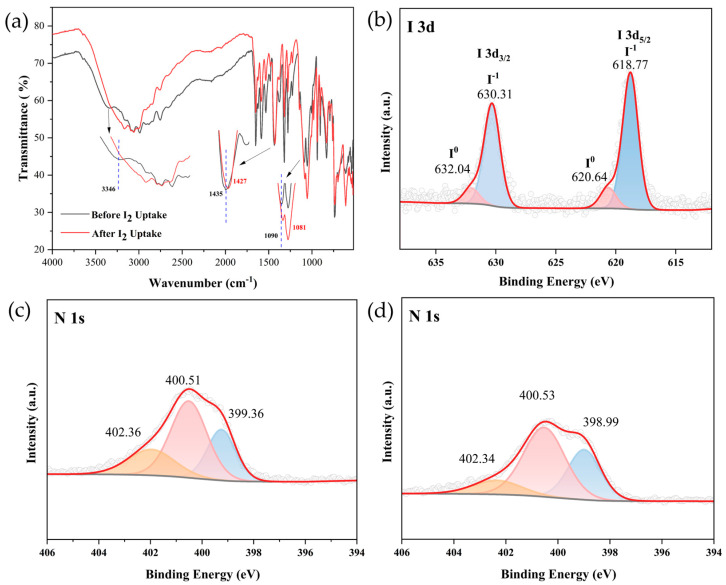
(**a**) IR spectra of compound **1** before and after uptake of I_2_ (inset: enlarged spectra showing band shifts at 1090 cm^−1^ and 1435 cm^−1^ and disappear at 3346cm^−1^). (**b**) XPS of I 3d of compound **1** after adsorption of iodine solution. (**c**) XPS of N 1s of compound **1** before uptake of dissolved I_2_. (**d**) XPS of N 1s of compound **1** after uptake of dissolved I_2_.

To confirm the hypothesis of iodine capture, Fourier transform infrared (FT-IR) (Figure 6a) and X-ray photoelectron spectroscopy (XPS) (Figure 6b–d) were performed on compound **1** before and after iodine adsorption. In Figure 6a, the contraction vibration absorption bands of *v*_C–S_, *v*_C–O_, *v*_C–N_, *v*_C=C-C_, *v*_N-H_, *v*_C=N_, *v*_-SH_, *v*_C-H_ and *v*_N-H_ could be clearly identified [36,37,38,39,40,41,42] (Table S6). After iodine uptake, the characteristic bands of 1090 cm^−1^ and 1435 cm^−1^ assigned to the C–H in-plane bending vibrations and C=C-C vibrations belonging to the aromatic rings were shifted, and the characteristic bands of 3346 cm^−1^ assigned to N-H belonging to benzimidazole groups disappeared. These changes implied that the electron-rich aromatic network of compound **1** might interact with electron-deficient iodine through a charge transfer process [43,44,45,46,47,48,49,50,51,52]. Meanwhile, consistent with the XRD results (Figure 3b), the peak shapes in the fingerprint region of FT-IR changed little, and the characteristic spectral bands were still clear, implying the stability of compound **1** before and after iodine adsorption. To understand the interaction between compound **1** and iodine more deeply, XPS data were performed on compound **1** both before and after the adsorption of iodine from an iodine/cyclohexane solution. As shown in Figure 6b, the peaks at I 3d could be divided into two groups. The stronger pair at 618.77 eV and 630.31 eV may be assigned to the I 3d_5/2_ and I 3d_3/2_ of I_3_^−^, while the weak pair at 620.64 eV and 632.04 eV are denoted to the I 3d_5/2_ and I 3d_3/2_ of I_2_ [53,54]. After I_2_ loading, the binding energy of N 1s signals shifted from 399.36 eV, 400.51 eV and 402.36 eV to 398.99 eV, 400.53 eV and 402.34 eV, respectively (Figure 6c,d). The binding energy of 399.36 eV is assigned to N atoms on the benzimidazole groups and the peak shows a more pronounced shift [55]. Based on the comprehensive analysis of the infrared (IR) and X-ray photoelectron spectroscopy (XPS) data for compound **1** before and after the adsorption process, it can be inferred that the aromatic molecules undergo electron transfer, resulting in the formation of positively charged molecular ions. Concurrently, iodine molecules accept electrons, thereby forming negatively charged ions. This electron transfer process facilitates the formation of stable charge-transfer complexes.

## 4. Materials and Methods

### 4.1. Synthesis of Compound ***1***

2-(2-benzimidazolyl)ethanethiol (L) was synthesized according to previously reported methods [43], NiCl_2_·6H_2_O was purchased from Adamas (Shanghai, China) and all other reagents were purchased from Shanghai HU Reagent & Chemical Reagent Group. All the reagents were used directly without purification. NiCl_2_·6H_2_O (0.119 g, 0.50 mmol) and 2-(2-benzimidazolyl)ethanethiol (L) (0.089 g, 0.50 mmol) were placed in a hydrothermal reactor containing 2 mL of acetonitrile, 8 mL of N,N-dimethylformamide and a trace amount of trimethylamine (0.05 mL). After being kept at 100 °C for three days, the mixture was cooled down to room temperature. Keeping the resulting brown solution undisturbed for three days, black crystals were obtained (Yield: 11.24%, based on L).

### 4.2. Characterization

Fourier transform infrared spectra (FTIR, Thermo Nicolet iS50, Thermo Fisher, Waltham, MA, USA) maps were tested in the range of 500–4000 cm^−1^ using a Thermo Nicolet iS50 instrument at room temperature with an acquisition background of air. Powder X-ray diffraction (PXRD, Miniflex 600, Akishima, Rigaku, Tokyo, Japan) patterns were obtained by scanning on a Miniflex 600 diffractometer using Mo *K*α rays (*λ* = - 0.71073 Å). X-ray photoelectron spectroscopy (XPS, Thermo Scientific K-Alpha, Waltham, MA, USA) studies were performed on an AXIS SUPRA Kratos system and the C ls line at 284.8 eV was used as a binding energy reference. TGA (TGA/DSC 1, Mettler Telodo, Zurich, Switzerland) was performed using a thermo plus EVO_2_ system in the range of 30–800 °C at a rate of 10 °C/min. UV-Vis spectra were recorded on an Agilent Cary 5000 spectrophotometer (UV-Vis, Agilent, Santa Clara, CA, USA).

### 4.3. Crystallography

Single crystal X-ray data were acquired by a microfocal spot small molecule diffractometer (Bruker D8 Venture, Thermo Fisher). Co K*α* rays (*λ* = 0.71073 Å) were used as the light source with a single photon detection efficiency of 0.99 and a cryogenic system temperature of ~200 K. Diffraction data reduction was performed by Apex3, and then the crystal structure was resolved and refined using olex2 1.5 software [56]. All nonhydrogen atoms were refined using the anisotropic refinement method and all hydrogen atoms were added to the geometrically optimal positions for isotropic refinement. Full crystallographic details in CIF format have been deposited in the Cambridge Crystallographic Data Centre (CCDC) under deposition number CCDC: 2418252 for compound **1**.

### 4.4. Iodine Adsorption Study

2-(2-benzimidazolyl)ethanethiol (L) was synthesized according to previously reported methods [57]. NiCl_2_·6H_2_O was purchased from Adamas (Shanghai, China) and all other reagents were purchased from Shanghai HU Reagent & Chemical Reagent Group. All the reagents were used directly without purification.

### 4.5. Iodine Adsorption Experiments

The iodine uptake behavior of compound **1** towards gaseous iodine and iodine in solution was investigated.

#### 4.5.1. Gaseous Iodine Adsorption of Compound **1**

Accurately weigh compound **1** (10 mg) in an uncapped glass vial and weigh the total mass of compound **1** and the vial, quickly place the vial into a sealed glass container containing an excess of iodine monomer at 70 °C, quickly place the sealed glass container into an oven at 70 °C, remove the vial after a certain period of time, weigh it after it has cooled down to room temperature and repeat the above operation until the adsorbent reaches saturation. until the adsorbent reaches adsorption saturation. The amount of iodine adsorbed is calculated using the following equation:

(1) $$Q_{t}=\frac{m_{2}-m_{1}}{m_{1}}$$

where *Q*_t_ denotes the amount of iodine adsorbed at a given time, *m*_1_ is the mass of compound **1** before iodine adsorption and *m*_2_ is the mass of compound **1** after iodine adsorption. The gaseous iodine adsorption equation was fitted using the pseudo-first order model (Equation (2)) and the pseudo-second-order model (Equation (3)). It was more consistent with the pseudo-second-order model and a set of parameters were obtained where *k*_2_ = 1.99757 × 10^−4^ g g^−1^ min^−1^ and *Q*_e_ = 2.48412 g g^−1^ and *R*^2^ = 0.98289.

(2) $$Q_{t}{=Q}_{e}{\;(1\;-\;e}^{k_{1}t})$$

(3) $$Q_{t}=\frac{k_{2}Q_{e}^{2}t}{{1+k}_{2}Q_{e}t}$$

where *k*_1_ is the quasi-primary adsorption rate constant, min^−1^, *k*_2_ is the quasi-secondary adsorption rate constant, g g^−1^ min^−1^, *Q*_e_ is the amount of iodine adsorbed at the equilibrium time, mg g^−1^ and *t* are the adsorption time, min.

#### 4.5.2. Iodine Adsorption in Solution of Compound **1**

The air-dried compound **1** (0.010 g) was immersed in 20 mL of iodine solution of cyclohexane (2.0 mmol/L). The iodine adsorption process was monitored by UV-VIS spectroscopy. The amount of iodine adsorbed can be calculated by Equation (4):

(4) $$Q_{t}=\frac{{(C}_{0}-C_{t})}{m}\;V$$

where *Q*_t_ represents the iodine absorption at a certain time, *C*_0_ and *C*_t_ represent the iodine concentration before and after adsorption, *m* represents the mass of compound **1** and *V* represents the volume of solution. The pseudo-first-order model (Equation (2)) and pseudo-second-order model (Equation (3)) were used to fit the kinetic adsorption equation of liquid iodine. The process was more consistent with the pseudo-second-order model, and a set of parameters was obtained, where *k* = 3.10857 × 10^−6^ g mg^−1^ min^−1^, *Q*_e_ = 614.60865 mg g^−1^ and *R*^2^ = 0.96219.

Compound **1** (0.010 g) was immersed in cyclohexane solutions of iodine at different concentrations (0.3, 0.5, 0.7, 0.9, 1.0, 2.0, 2.0 mmol/L) at room temperature. The iodine adsorption process was monitored by UV-Vis spectroscopy. The amount of iodine adsorbed was calculated by Equation (4). The Langmuir equation (Equation (5)) and Freundlich equation (Equation (6)) were used to fit the isothermal adsorption model, respectively. The process was more consistent with the Langmuir equation and a set of parameters was obtained where *K*_L_ = 0.00133 L mg^−1^ and *R*^2^ = 0.99891.

(5) $$Q_{e}=\;\frac{Q_{m}K_{L}C_{e}}{{1+K}_{L}C_{e}}$$

(6) $$Q_{e}{=K}_{F}C_{e}^{\frac{1}{n}}$$

where *K*_L_ is Langmuir’s constant, L mg^−1^, *K*_F_ is the adsorption capacity, mg^1−n^ L^n^ g^−1^, *n* is the Freundlich model constant.

#### 4.5.3. Iodine Release and Recyclability of Compound **1**

I_2_@**1** (10 mg) was immersed in methanol (20 mL) to release adsorbed iodine. Compound **1** after iodine release was placed in a vacuum drying oven for 24 h. I_2_ adsorption experiment was re-performed on regenerated compound **1** to evaluate the cyclic adsorption performance of the sample. After three cycles, about 90% of the I_2_ adsorption capacity was retained.

## 5. Conclusions

In summary, a three-dimensional framework of the benzimidazole-based compound **1** was successfully constructed through hydrogen bonding and *π–π* stacking interactions. Owing to its *π*-electron-rich benzimidazole moiety, compound **1** demonstrates exceptional adsorption capabilities for both gaseous and dissolved iodine, achieving adsorption capacities of 2.08 g g−1 and 560 mg g−1, respectively. Mechanistic investigations reveal that the adsorption process is predominantly governed by charge transfer interactions between the aromatic network of compound **1** and iodine molecules. Specifically, the electron-rich aromatic network serves as an electron donor, transferring electrons to the electron-deficient iodine molecules to form a stable charge-transfer complex. The adsorption kinetics and isothermal adsorption models further support the predominance of chemisorption in this process. Given its high iodine adsorption efficiency and well-defined adsorption mechanism, compound **1** exhibits significant potential for radioactive iodine capture, offering valuable insights and practical guidance for the development of advanced iodine adsorption materials.

## Data Availability

All data related to this study are presented in this publication.

## Supplementary Materials

The following supporting information can be downloaded at https://www.mdpi.com/article/10.3390/molecules30050989/s1. Table S1: Crystal structure and refinement data for compound **1**; Table S2: Parameter fitting of the kinetic model for the adsorption of gaseous iodine by compound **1**; Table S3: Parameter fitting of the kinetic model for the adsorption of compound **1** on liquid iodine; Table S4: Parameter fitting of thermodynamic model for adsorption of compound **1** on liquid iodine; Table S5: A review of the iodine adsorption capacity of adsorbent materials in gases and solutions; Table S6: Analysis of the peak positions in the infrared spectra of compound **1**; Figure S1: The structure of ligand L. Figure S2: I_2_ vapor adsorption unit; Figure S3: Photograph showing the color of the I_2_-cyclohexane solution at adsorption equilibrium when 10 mg of compound **1** is immersed in the I_2_-cyclohexane solution; Figure S4: Photograph showing the change in color of the crystals of compound **1** before and after adsorption of dissolved I_2_; Figure S5: Photograph showing the release of I_2_ from I_2_@**1** in methanol; Figure S6: XPS spectrum of compound **1**.
