# Supplementary material for: Synthesis, Structure and Iodine Adsorption Properties of a Ni Cluster-Based Supramolecular Framework
Source: Molecules. 2025 Feb 21;30(5):989. doi: 10.3390/molecules30050989 (PMC11901886; doi:10.3390/molecules30050989)
Supplement: Supplementary file 1 [file molecules-30-00989-s001.zip › molecules-3422259-supplementary.pdf]

# Synthesis, Structure and Iodine Adsorption Properties of a Ni Cluster-Based Supramolecular Framework

**Jing-Yi Qiu [1,3], Lin-Xia Tang [1,3], Zi-Ang Nan [3], Lu-Yao Liu [3], Qing Li [3], Wei Wang [3], Zhu Zhuo [3,*], Dong-Wei Zhang [2,*], You-Gui Huang [3], Liang-Liang Zhang [4,*]**

[1] College of Chemistry and Materials Science, Fujian Normal University, Fuzhou, Fujian, 350002, China

[2] School of Microelectronics, Northwestern Polytechnical University, Xi'an 710072, China

[3] Xiamen Key Laboratory of Rare Earth Photoelectric Functional Materials, Xiamen Institute of Rare Earth Materials, Haixi Institutes, Chinese Academy of Sciences, Xiamen, Fujian, 361021, China

[4] Strait Institute of Flexible Electronics (SIFE, Future Technologies), Fujian Normal University and Strait Laboratory of Flexible Electronics (SLoFE), Fuzhou, 350117, China

**Table S1.** Crystal structure and refinement data for compound **1**.

| **Compound** | **1** |
|---|---|
| Formula | $C_{101}H_{90}Cl_3N_{26}Ni_5O_{10}S_{6.5}$ |
| Formula weight | 2436.27 |
| Temperature/K | 200.00 |
| Crystal system | monoclinic |
| Space group | $C2/c$ |
| $a$/Å | 18.0172(16) |
| $b$/Å | 27.317(3) |
| $c$/Å | 21.9914(18) |
| $\alpha$/° | 90 |
| $\beta$/° | 99.817(2) |
| $\gamma$/° | 90 |
| *Volume*/Å$^3$ | 10665.3(16) |
| $Z$ | 4 |
| $\rho_{cal.}$/(g cm$^{-3}$) | 1.517 |
| $\mu$/mm$^{-1}$ | 1.140 |
| $F$(000) | 5012.0 |
| $R_{int}$ | 0.1294 |
| *GOF* | 1.016 |
| $^aR_1$, $^bwR_2$ [I>2$\sigma$($I$)] | $R_1$ = 0.0857, $wR_2$ = 0.2170 |
| $^aR_1$, $^bwR_2$ [all data] | $R_1$ = 0.1825, $wR_2$ = 0.2741 |
| CCDC number | 2418252 |

**Table S2.** Parameter fitting of the kinetic model for the adsorption of gaseous iodine by compound **1**.

| $Q_{e,exp}$ ($g\ g^{-1}$) | **Pseudo first-order Kinetic Model Fitting** | | | **Pseudo second-order Kinetic Model Fitting** | | |
|---|---|---|---|---|---|---|
| | $k_1$ / ($min^{-1}$) | $Q_{e,exp}$ / ($g\ g^{-1}$) | $R^2$ | $k_2$ / ($g\ g^{-1}\ min^{-1}$) | $Q_{e,exp}$ / ($g\ g^{-1}$) | $R^2$ |
| 2.08 | $4.81051\times10^{-4}$ | 1.98927 | 0.97077 | $1.99757\times10^{-4}$ | 2.48412 | 0.98289 |

**Table S3.** Parameter fitting of the kinetic model for the adsorption of compound **1** on liquid iodine.

| $Q_{e,exp}$ ($mg\ g^{-1}$) | **Pseudo first-order Kinetic Model Fitting** | | | **Pseudo second-order Kinetic Model Fitting** | | |
|---|---|---|---|---|---|---|
| | $k_1$ / ($min^{-1}$) | $Q_{e,exp}$ / ($mg\ g^{-1}$) | $R^2$ | $k_2$ / ($mg\ g^{-1}\ min^{-1}$) | $Q_{e,exp}$ / ($mg\ g^{-1}$) | $R^2$ |
| 562.27445 | 0.00184 | 509.33752 | 0.9471 | $3.10857\times10^{-6}$ | 614.60865 | 0.96219 |

**Table S4.** Parameter fitting of thermodynamic model for adsorption of compound **1** on liquid iodine.

| Temperature (K) | **Langmuir Model** | | | **Freundlich Model** | | |
|---|---|---|---|---|---|---|
| | $K_L$ / ($L\ mg^{-1}$) | $Q_{max}$ / ($mg\ g^{-1}$) | $R^2$ | $K_F$ / ($mg^{1-n}\ L^n\ g^{-1}$) | $n$ | $R^2$ |
| 298 | 0.00133 | 1392.12174 | 0.99891 | 5.42883 | 1.33801 | 0.99424 |

**Table S5.** The comparison of $I_2$ adsorption abilities for different materials.

| **Adsorbents** | **$I_2$ vapor (g $g^{-1}$)** | **Adsorption of $I_2$ in solution (g $g^{-1}$)** | **Ref.** |
|---|---|---|---|
| $[Zn_3(BTC)_2(TIB)_2]^a$ | 0.04 | / | 22 |
| $[Cu_4I_4(L_3)]$ | 0.14 | / | 23 |
| $[Ni(L_2)_2Cl_2]^a$ | 0.22 | / | 24 |
| $[Ni(44pba)_2]^a$ | 1.10 | / | 25 |
| Th-TATAB[b] | 0.225 | 0.075 | 26 |
| $[DMA][In(TDC)_2]$ | / | 0.100 | 27 |
| $[Zn_2(\mu_4\text{-}ao_2btc)(\mu\text{-}pbix)_2]_n$ | / | 0.180 | 28 |
| HKUST-1@ PVDF | / | 0.225 | |
| **Compound 1** | **2.08** | **0.560** | **This work** |

**Table S6.** Analysis of the peak positions in the infrared spectra of compound **1**.

| Functional group | Wavenumber ($cm^{-1}$) |
|---|---|
| C-S | ~610 |
| C-H | ~1090 |
| C-O | ~1278 |
| C-N | ~1320 |
| C=C-C | ~1435 |
| N-H | ~1531 |
| C=N | ~1651 |
| -SH | ~2162 |
| C-H | ~3058 |
| N-H | ~3346 |

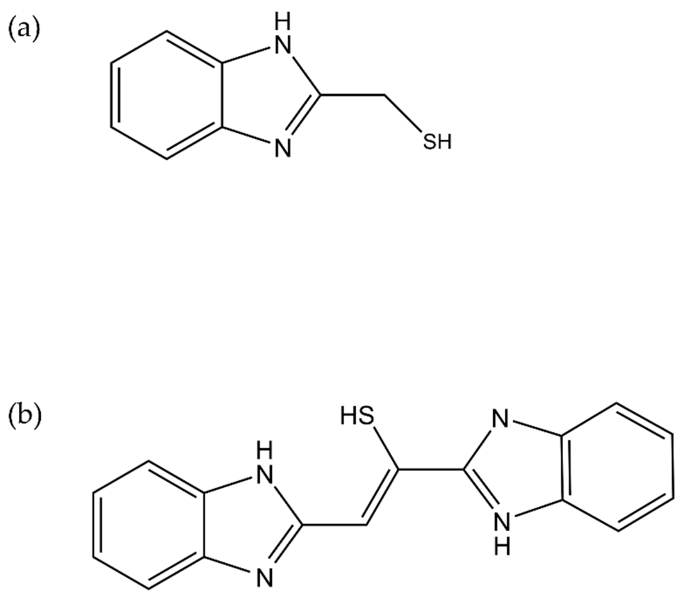


**Figure S1.** (**a**) The structure of ligand L. (**b**) The structure of ligand $L_1$.

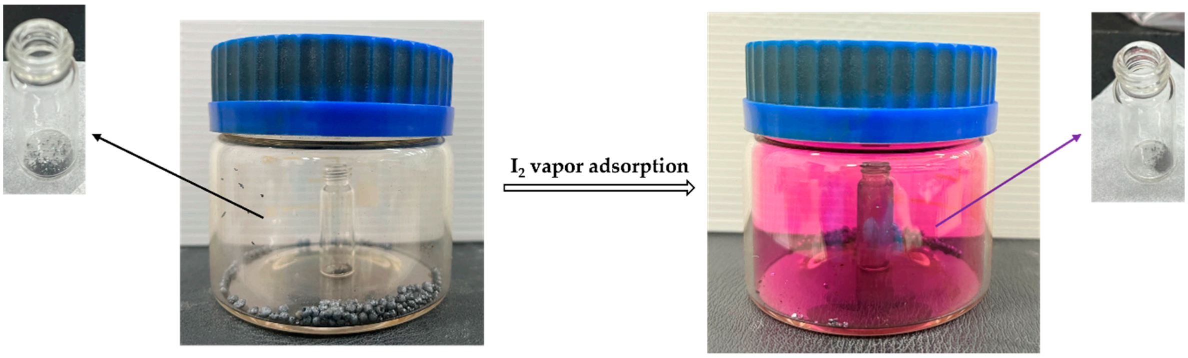


**Figure S2.** $I_2$ vapor adsorption unit.

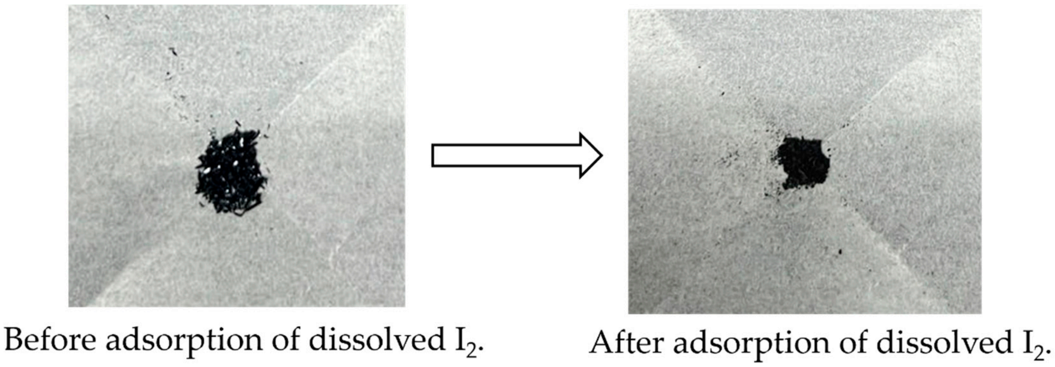


**Figure S3.** Photograph showing the change in color of the crystals of compound **1** before and after adsorption of dissolved $I_2$.

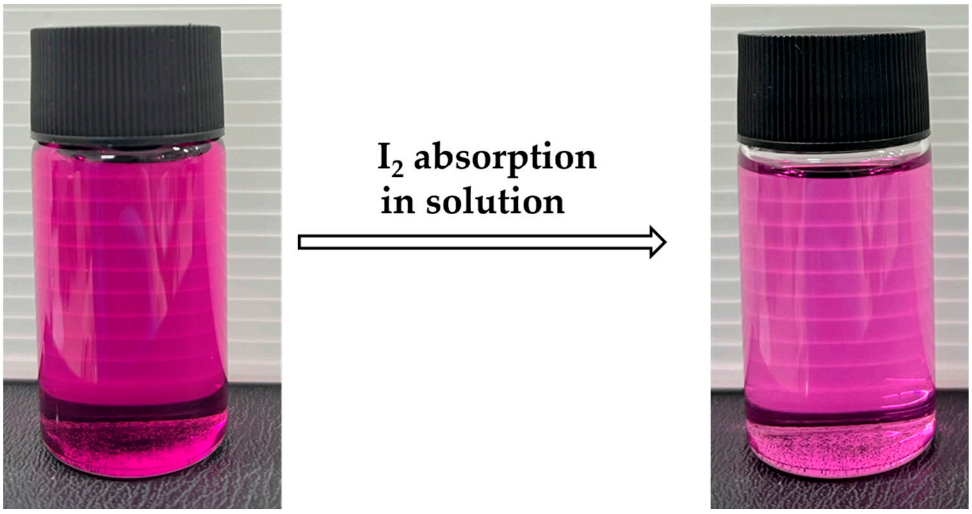


**Figure S4.** Photograph showing the color of the $I_2$-cyclohexane solution at adsorption equilibrium when 20 mg of compound **1** is immersed in the $I_2$-cyclohexane solution.

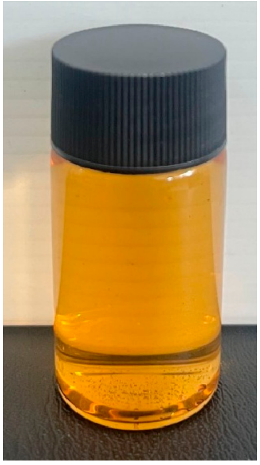

**Figure S5.** Photograph showing the release of $I_2$ from $I_2$@**1** in methanol.

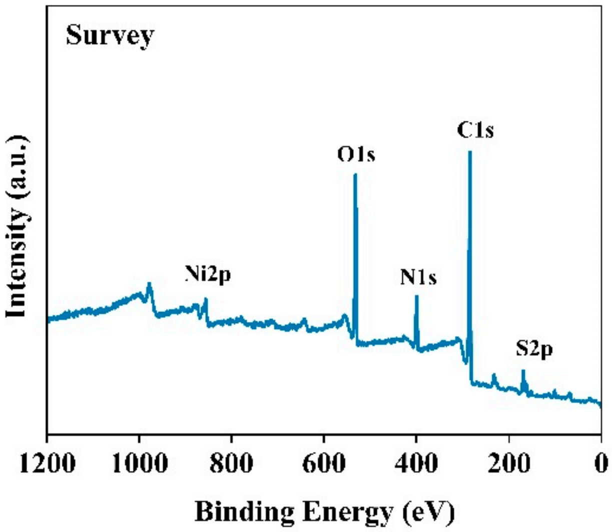


**Figure S6.** XPS spectrum of compound **1**.
